# Memory encoding for new information, not autobiographical memory load, is linked with age-related differences in subjective time passage over the past decade

**DOI:** 10.3758/s13421-026-01879-1

**Published:** 2026-04-07

**Authors:** Alice Teghil, Sebastian Wittmann, Adele Lifrieri, Sophia Saad, Maddalena Boccia, Marc Wittmann

**Affiliations:** 1 Department of Psychology, Sapienza University of Rome, Via Dei Marsi 78, 00185 Rome, Italy; 2 Cognitive and Motor Rehabilitation and Neuroimaging Unit, IRCCS Fondazione Santa Lucia, Rome, Italy; 3 Institute for Frontier Areas of Psychology and Mental Health, Freiburg, Germany

**Keywords:** Time perception, Passage of time, Age, Cognitive functioning, Autobiographical memory

## Abstract

The widely replicated finding that time passage over the past decade is perceived as faster by older adults was the focus of this study. We investigated potential factors associated with this effect, examining the role of autobiographical memory and cognitive functioning. One hundred and twenty individuals aged 20–91 years were assessed on subjective time perception for the preceding decade and year, the quantity and significance of autobiographical memories from those periods, and overall cognitive status. Results confirmed the specific age-related effect concerning a faster time passage over the past decade. However, no significant association was found between perceived time passage over the past decade and either the number or subjective value of memories. Contrary to assumptions, older adults reported more vivid and meaningful recollections. Instead, reduced cognitive functioning, and specifically lower ability to form new memories as assessed through delayed memory recall, emerged as significantly linked to faster time perception with older age. Findings suggest that age-related cognitive decline leading to reduced ability to encode novel memories, rather than diminished autobiographical memory content, is a critical factor in the subjective experience of time compression in older adults.

**Supplementary Information:**

The online version contains supplementary material available at 10.3758/s13421-026-01879-1.

## Introduction

Many people believe that time feels like it moves faster as we get older, and this idea is commonplace. While this phenomenon of perceived temporal acceleration is generally acknowledged among laypersons, robust empirical support has only begun to solidify over the past 2 decades. Early evidence emerged from a seminal study conducted in 2005 involving approximately 500 Austrian and German participants, which identified faster subjective speed of time passage in older adults when participants were asked, “How fast did the past 10 years pass for you?” (Wittmann & Lehnhoff, 2005). This initial finding, focusing on the retrospective evaluation of a decade, has since been replicated in multiple subsequent studies, consistently demonstrating a similar age-related pattern.

For instance, two further studies conducted with German samples reported positive correlations between age and perceived speed of time passage over both the previous 5 years (Winkler et al., 2017) and 10 years (Wittmann et al., 2015). The original 2005 findings have also been corroborated in cross-cultural contexts, with comparable results observed among participants from the Netherlands and New Zealand (Friedman & Janssen, 2010) as well as Japan (Janssen et al., 2013). Further extending this line of inquiry, research with a French Canadian cohort revealed the strongest age effects when participants reflected on the past 3, 5, and 10 years (Gagnon-Harvey et al., 2021).

Collectively, these studies suggest that, at least among individuals in industrialized nations, age-related differences in the subjective speed of time become increasingly pronounced when reflecting on longer temporal spans—specifically the last 3 to 10 years. However, it is important to note that while the effect is consistently replicated, its magnitude remains modest, with correlation coefficients typically ranging from 0.2 to 0.3 across studies. Moreover, the effect is specific to the period of the last 3 to 10 years; no significant associations are observed for shorter periods, such as the previous week, month, or year (Droit-Volet & Wearden, 2015).

One explanation for why older adults feel that time has been passing faster compared with younger adults has to do with how we remember our lives. As we age, our daily routines tend to become more repetitive and predictable. Because of this, we experience fewer new or exciting events, which means that fewer memorable moments are stored in our autobiographical memory. As a result, when we look back, these later periods in life seem less full and shorter (Bruss & Rüschendorf, 2010; Draaisma, 2004; Flaherty & Meer, 1994). This idea fits with the concept of the "reminiscence bump"—a well-known pattern in which people can recall more personal memories from the ages of about 15 to 30 than from other stages of life (Kawasaki et al., 2011).

Research shows that the number and emotional intensity of experiences we have in a given time period affect how long that period feels in hindsight. Studies have found that when people have more frequent and emotionally meaningful experiences—for short intervals of time, like seconds or minutes—they later remember those time spans as having lasted longer (Block & Zakay, 1997; Block et al., 2000; Droit-Volet et al., 2018). In short, the more varied and meaningful the experiences during a certain time, the longer it tends to feel when we think back on it.

The valence of experienced events also affects retrospective duration judgments. Stimuli with a positive valence, such as amusing movie clips, are rated in hindsight as having lasted shorter than those with a negative valence (fearful movie clips; Pollatos et al., 2014). Similarly, in ecological settings, time passage has been found to be retrospectively rated as slower during fearful events (Campbell & Bryant, 2007). These effects are consistent with evidence that emotional valence and personal significance affect the way long-term memories for events are encoded and retrieved (see Holland & Kensinger, 2010, for a review). For example, arousing and emotionally meaningful experiences are often remembered with high accuracy and vividness (see Buchanan, 2007, for a review). Thus, emotional valence, significance, and possibly vividness of memories, may also have an impact on the perception of time passage for long time spans. Notably, older age has been associated with increased propensity to remember positive experiences (Ford et al., 2016, 2018), as well as with a reduction of the effect of emotion on accuracy, but not on vividness of recalled memories (Faul et al., 2025), further suggesting that these factors may have a role and in explaining age-related differences in time experience.

Studies assessing students’ lifetime periods showed that a larger amount of processed memory items is related to a felt slower passage of time over the past year (Landau et al., 2018) and the previous 5 years (Kosak et al., 2019). Results with these student populations are not unequivocal. In a recent series of four studies, people did not report actual individual memories over the past year but had to just indicate the specific number of outstanding events (Ryu et al., 2024). This measure of reported memory number correlated positively with the subjective pace of time over the past year in three out of four studies. Contrary to expectation, the larger the number reported the faster time passage. In only two out of these four studies, reported routine over the past year correlated with the subjective pace of time in the expected way, albeit with a small coefficient: the more routine, the faster the pace of time. In another study more routine predicted a faster passage of time and more life experiences, predicted a slower passage of time (Winkler et al., 2017). These effects of routine and number of new life experiences fit the hypothesis regarding memory function as being responsible for the aging effect. However, despite the body of research examining the role of memory load in the subjective experience of time across the lifespan, no single study to date has directly demonstrated that a decline in autobiographical memory recall is associated with faster perception of time passage with increasing age.

Another possible explanation for the consistently observed effects of aging in the perception of time involves age-related cognitive decline. Core executive functions—such as working memory, fluid reasoning, and attentional control—are known to decline progressively with age (Christensen, 2001; Pütz et al., 2012a, 2012b; Fig. 3), and these declines have been linked to reduced accuracy and precision in timing tasks involving intervals of up to several seconds (Baudouin et al., 2019; Lamotte & Droit-Volet, 2017; Mioni et al., 2020; Pütz et al., 2012a, 2012b; Ulbrich et al., 2009). Given that executive functions are critical for perceptual processing (Turgeon et al., 2016), it is reasonable to assume that impairments in these domains would influence how time is experienced.

In particular, the slowing of cognitive processing speed commonly associated with physiological aging (Baudouin et al., 2019; Christensen, 2001; Pütz et al., 2012b) may reduce the amount of sensory information processed within a given time frame. This reduction in the density of perceived events could result in fewer moments being encoded, and lead to the retrospective impression that time has passed more quickly (Bejan, 2019). Aging is also commonly associated with a reduction in episodic memory encoding (Balota et al., 2000; Cansino, 2009), which could similarly result in less available events in memory, and faster perceived passage of time when judged retrospectively.

Despite this theoretical framework, no empirical study to date has directly examined whether cognitive decline contributes to the perception of time passage over extended intervals, such as the past 10 years. This represents a significant gap in the literature and warrants further investigation.

The present study aimed to investigate the relationship between age-related differences in subjective time passage, autobiographical memory recall and physiological cognitive decline. Based on literature showing that the subjective impression that time has passed faster in people with older age is particularly apparent when considering long temporal spans (Friedman & Janssen, 2010; Gagnon-Harvey et al., 2021; Wittmann & Lehnhoff, 2005; Wittmann et al., 2015; Winkler et al., 2017), we focused our investigation over the period of the past 10 years. As a control to test for the specificity of any effect, subjective time passage and autobiographical memory were further assessed for the period spanning the past year, for which a positive association between age and perceived temporal speed was not expected (Droit-Volet & Wearden, 2015; Friedman & Janssen, 2010; Gagnon-Harvey et al., 2021; Wittmann & Lehnhoff, 2005; Wittmann et al., 2015). The relationship between age-related differences in subjective time passage, autobiographical memory recall and physiological cognitive decline was thus addressed over the two time periods (the past 10 years and the past year) in a set of confirmatory analyses.

## Materials and methods

### Participants

A total of 120 participants was included in this study, comprising 60 men and 60 women. The participants were questioned across different cultural settings, with 60 participants from Italy (15 from Rome, 45 from Southern Italy), and 60 participants from two German-speaking countries (41 participants from the Freiburg area, Germany, two from a Northwest German town, and 17 from Zürich, Switzerland). Participants were categorized into age groups: 20–29, 30–39, 40–49, 50–59, 60–69, 70 plus (age range: 70–91). In both the Italian- and German-speaking samples, five men and five women participants were recruited for each age group. Thus, the final sample included 120 participants, with 20 participants (10 Italian-speaking, 10 German-speaking) for each age group. Participants were recruited by word-of-mouth. Recruitment in each language group was carried out until the target sample size and proportion of men and women participants per age group were reached—after which, recruitment focused on the enrolment of participants with the required demographic characteristics. To be eligible, participants had to be at least 20 years old, exhibit no signs of mental illness, dementia, or Alzheimer’s disease, and report no previous history of neurological or psychiatric disorders. They also had to be fluent in either Italian or German. All included participants scored above the cut-off in a cognitive screening test (the Tele-Global Examination of Mental State; Montemurro et al., 2023, see below).

Concerning educational background, three participants had the basic level of education (primary school), nine had completed secondary school, 61 had graduated in high school or equivalent, and 47 had a university or college degree or a higher qualification (e.g., doctoral degree). Demographic information on participants is reported in Table 1.

**Table 1:** Demographic information on participants, according to language group (Italian-speaking, German-speaking) and for the total sample (*N* = 120)

	Age		Education		Tele-GEMS	
Age group	*M*	*SD*	*M*	*SD*	*M*	*SD*
Italian-speaking						
1	23.40	1.90	16.4	1.78	91.57	5.69
2	32.40	2.22	14.9	2.18	87.54	4.17
3	44.60	2.32	18.0	3.83	87.00	6.78
4	55.40	2.67	15.5	4.22	88.69	4.78
5	64.20	3.05	15.6	5.23	84.93	6.38
6	79.80	4.83	10.0	4.55	74.96	6.62
German-speaking						
1	23.40	3.69	15.10	3.11	94.37	2.80
2	33.70	2.50	22.20	3.22	94.43	2.57
3	44.20	2.53	19.50	4.41	90.53	5.24
4	55.20	2.70	18.65	1.94	90.91	5.97
5	65.00	2.71	19.35	4.14	89.63	7.06
6	76.90	4.65	16.10	3.26	84.42	7.18
Total sample						
1	23.40	2.85	15.75	2.55	92.97	4.60
2	33.05	2.39	18.55	4.61	90.98	4.89
3	44.40	2.37	18.75	4.09	88.76	6.17
4	55.30	2.62	17.08	3.58	89.80	5.39
5	64.60	2.84	17.48	4.98	87.28	6.98
6	78.35	4.85	13.05	4.96	79.69	8.29

For each age group (1 = age group 20–29; 2 = age group 30–39; 3 = age group 40–49; 4 = age group 50–59; 5 = age group 60–69; 6 = age group > 70), mean and standard deviation of age, years of education and score on the Tele-Global Examination of Mental State (Tele-GEMS) are reported

To define sample size, an a priori power analysis was performed using Jamovi (Version 2.3.19.0), aiming for 90% power, with alpha = 0.05. Cohen’s *d* (1.69) was derived comparing scores on the item of the Subjective Time Questionnaire (Wittmann & Lehnhoff, 2005; Wittmann et al., 2015) assessing the perceived speed of time over the past 10 years between 17 healthy older adults (age range: 65–82 years) which participated in a previous study (Teghil et al., 2023) and 14 healthy volunteers (six women) aged 19–30 (*M* = 24.14 years, *SD* = 2.96). The resulting sample size was of nine individuals per group; thus, we enrolled 10 participants for each age group per language (*n* = 60; see above). With an overall sample size of 120 (60 per language) we expected to have enough power for the adjustment of significance levels for multiple testing.

Participants from Freiburg received a compensation of 10 euros and those from Zurich 10 Swiss francs. The study was approved by the local Ethics Committees of Sapienza University of Rome (for Italian participants, Prot. n. 0000512, 18/03/2022) and the Institute for Frontier Areas of Psychology and Mental Health (for German speaking participants; IGPP_2024_05). All participants provided written informed consent prior to data collection. No part of the study was preregistered. The dataset used in all reported analyses may be accessed online (https://osf.io/t65k9/?view_only=25bf94f7cca6481fb1cbc0b03d9e0c9c).

### Subjective passage of time

All participants performed the Subjective Time Questionnaire (STQ; Wittmann & Lehnhoff, 2005; Wittmann et al., 2015; the complete set of questions included in the questionnaire can be found in Supplementary materials, File S1). The questions included in this study were the retrospective judgment of past time intervals asking how quickly the past 10 years and the past year have seemed to pass. The response options ranged from 1 (*very slowly*) to 5 (*very fast*).

### Autobiographical memory retrieval

An adapted version of the Autobiographical Fluency Task (AFT; Conti et al., 2024) was used to quantitatively evaluate participants' ability to access personal memories over specific time periods. In this task, participants are asked to recall as many specific autobiographical events as possible within 90 s. Instructions specify that they have to provide a label for the events, without further adding details; thus, no detailed recollection of accessed events is required. This was done for two lifetime periods in the present study: First, participants had 90 s to recall memories from the past year, and then they were asked to recall memories from the past 10 years, excluding the past 12 months. Afterward, participants rated each memory item on a scale for (1) how positive the event was perceived to be at the time it occurred and (2) how positive it was considered now. They did the same for how (3) negative the event was at the time and (4) now, as well as (5) how significant they considered the event at the time and (6) now. The final question asked (6) how vivid each memory was. For each of these questions, the answer scale ranged from 1 (*not at all*) to 5 (extremely); that is, answer ranges varied between not at all positive/negative/significant/vivid to extremely positive/negative/significant/vivid, respectively. The AFT aims to measure the ease with which individuals can access personal memories and to explore the relationship between memory fluency and cognitive/emotional variables. The average value across subjects was calculated for each of the different aspects. The total number of items provided for each life period (last year, past 10 years) was calculated as an index of autobiographical fluency.

### Assessment of cognitive functioning

Global cognitive functioning was assessed using the Tele-Global Examination of Mental State (Tele-GEMS), originally devised to evaluate participants’ cognitive and mental state remotely (Montemurro et al., 2023). In this study the participants were questioned by the interviewer sitting next to them. This standardized assessment covers various cognitive domains, including orientation, immediate memory, working memory, spatial representation, language, delayed memory, attention, executive functions and the pragmatics of language. Participants undergo 10 tasks designed to measure cognitive abilities, with their performance scored as sum across categories.

### Procedure

The experimental procedure took place in a single session, lasting approximately 45 to 60 min. All tasks and questionnaires were administered in person. Tasks were completed following a fixed order, that is, first the Tele-GEMS, then the STQ, and lastly the AFT. This order was chosen to ensure that participants’ global cognitive functioning was assessed at the beginning of the session, thus allowing is to detect any suspect of mental deterioration or dementia. Also, the administration of the AFT after the STQ was aimed to rule out influences of performance on the AFT on subjective time judgments (see Kosak et al., 2019, for a discussion of this issue). Participants also performed the Autobiographical Recollection Test (ART; Berntsen et al., 2019) and the Survey of Autobiographical Memory (SAM; Palombo et al., 2013), which were administered after the STQ and were not considered in the present study since they were not part of confirmatory hypotheses on the subjective passage of time.

## Statistical analyses and results

As data did not generally meet the assumptions of parametric analyses (results of Kolmogorov–Smirnov tests for 85% of assessed variables ranged between 0.092 and 0.293, *p* <.05), robust or non-parametric statistical methods were used for all analyses.

A comparison between language groups (Italian- and German-speaking) showed that the German-speaking group had, on average, significantly more years of education than the Italian-speaking group (*U* = 1,032,* p* <.001), as well as higher scores on the Tele-GEMS (*U* = 1,064, *p* <.001) (means and standard deviations are reported in Table 1). Scores on the items of the STQ assessing the perceived speed of time did not differ between language groups, neither for the past 10 years (Italian-speaking: *M* = 3.47, *SD* = 0.965; German-speaking: *M* = 3.63, *SD* = 1.02; *U* = 1,627.5, *p* =.343) nor the past year (Italian-speaking: *M* = 3.72, *SD* = 1.043; German-speaking: *M* = 3.88, *SD* = 0.804; *U* = 1,684, p =.511). Also, education was not significantly correlated with either subjective speed of time scores for the past 10 years (rho = 0.006, *p* =.952) nor the past year (rho =  − 0.1, *p* =.275). Thus, data from Italian- and German-speaking participants were aggregated in following analyses.

### Association between experience of time passage over the past 10 years, age, autobiographical memory recall, and cognitive functioning

In a first set of confirmatory analyses, Spearman correlation coefficients were calculated between scores on the item of the STQ assessing the experienced speed of time passage over the past 10 years, and (1) participants’ age, treated as a continuous variable; (2) the number of recalled autobiographical events over the past 10 years, as assessed by the AFT; (3) total score on the Tele-GEMS; and (4) self-rated positivity (negativity ratings were reversed and summed to positivity ratings to derive an integrate score), significance and vividness of the recalled autobiographical events over the same time period (*n* = 8 correlations). As ratings of positive valence and significance of the recalled autobiographical events were significantly different between “the time the event occurred” and “now” (see Supplementary materials, Table S1, for descriptive statistics), they were considered separately in all correlation analyses.

**Fig. 1:**
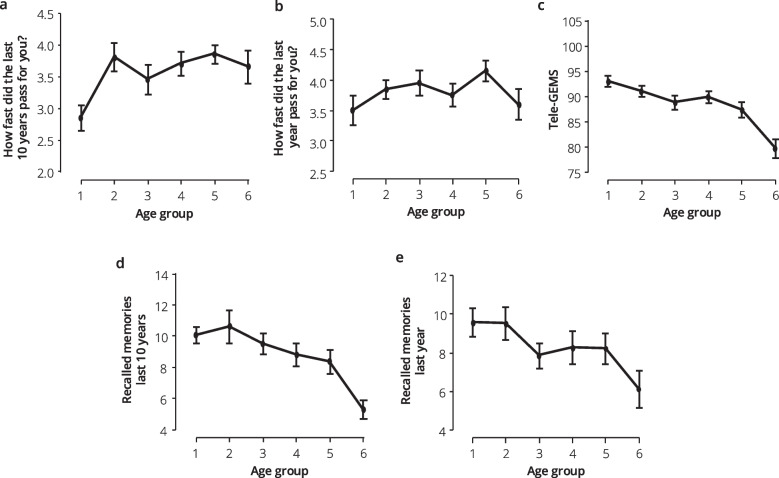
Means and standard errors over the different age groups. **a** Experienced speed of time over the past 10 years. **b** Experienced speed of time over the past year. **c** Total Tele-GEMS score. **d** Number of recalled memories over the past 10 years. **e** Number of recalled memories over the past year. 1 = age group 20–29; 2 = age group 30–39; 3 = age group 40–49; 4 = age group 50–59; 5 = age group 60–69; 6 = age group > 70

As expected, a significant positive correlation was observed between the question concerning the experienced speed of time over the past 10 years and participants’ age (*p* =.004; Fig. 1a). The experienced speed of time over the past 10 years further correlated negatively with the total Tele-GEMS score (*p* =.004). Both correlations survived correction for multiple comparisons (adjusted alpha level set at .05/8 =.006). There was no significant correlation between the subjective speed of time over the past 10 years and the number of recalled memories for the same time period (*p* =.305), nor with their subjective ratings (“positive then”: *p* =.115; “positive now”: *p* =.084; “significant then”: *p* =.592; “significant now”: *p* =.778; vividness: *p* =.546). Correlation coefficients are reported in Table 2 (Column 1). Means and standard deviations according to age groups are reported in Supplementary materials (Table S2).

**Table 2:** Column 1 shows Spearman correlation coefficients between subjective passage of time over the past ten years and age (in years), total Tele-GEMS score, and autobiographical memory retrieval over the past ten years

Variable	*M*	*SD*	1	2
1. How fast did the last 10 years pass for you?	3.55	1.00	—	
2. Age	49.85	18.90	.26**	—
3. Tele-GEMS	88.25	7.39	−.26**	−.49***
4. Number of recalled memories	8.77	3.72	−.09	−.42***
5. Positive then	7.87	1.37	.14	.08
6. Positive now	7.98	1.40	.16	.02
7. Significant then	4.41	0.54	.05	.30***
8. Significant now	4.10	0.80	−.03	.27**
9. Vividness	4.36	0.70	.06	.34***

Column 2 shows Spearman correlation coefficients between age (in years), total Tele-GEMS score, and autobiographical memory retrieval over the past 10 years. *p <.05. **p <.01. ***p <.001 (two-tailed)

It has to be noted that participants in the AFT were given 90 s to retrieve from memory as many personal events as possible which had happened during a specific time period (see Materials and Methods). It is conceivable that, when reflecting on perceived time passage over extended periods such as the past 10 years, individuals only base their judgment on events which are more readily recollected. To explore this possibility, correlation coefficients were calculated between the question concerning the experienced speed of time over the past 10 years and subjective ratings of events, considering only the first three events retrieved in the AFT for the same time period. No correlation survived correction for multiple comparison. Results are reported in Supplementary materials (Table S3).

As the positive association between age and the subjective speed of time over the past 10 years seemed to be particularly apparent for the youngest age group (20–29 years), of which participants showed especially low ratings (Fig. 1a), in a further exploratory analysis we examined if excluding this group from analyses could affect the results. When excluding the youngest age group, the correlation between age and the subjective speed of time over the past 10 years was no longer significant (*N* = 100, rho = 0.05, *p* =.617), suggesting that the observed effect in the present sample was at least partially driven by a relatively slower subjective passage of time in young adults as compared to the older age groups.

Finally, we explored a possible nonlinear association between age and the subjective speed of time over the past 10 years, testing a quadratic regression model with age as a predictor of subjective time passage scores. The quadratic model was significant, *F*(2,117) = 5.431, *p* =.006, adjusted *R*-squared =.069. Age had a significant positive linear association with the perceived speed of time over the past 10 years (β = 0.069, *SE* = 0.027, *t* = 2.57, *p* =.011), while the quadratic term was negative (β =  − 0.00056, *SE* = 0.00026, *t* =  − 2.18, *p* =.032). Thus, the relation was primarily linear, with a small but significant quadratic component suggesting a deceleration at older ages.

### Control analysis on the experienced time speed over the past year

To confirm the specificity of the positive association between older age and subjective time passage for the period of the past 10 years (vs. relatively faster time passage; see the Introduction), a correlation matrix analogue to that described above was calculated, assessing the association of the experienced speed of time passage over the past year with age (continuous variable), total score on the Tele-GEMS, number and subjective ratings of recalled autobiographical events (separately for the “time the event occurred” and “now”) in the AFT over the past year (*n* = 8 correlations).

No significant correlation was observed with age (*p* =.419; Fig. 1b), nor with the total Tele-GEMS score (*p* =.054) or the number of recalled memories over the past year (*p* =.657). The perceived speed of time correlated positively with the mean reported positive valence of events at the time they occurred (“positive then”; *p* =.003); a positive correlation was also observed with the mean positive valence of events “now” (*p* =.012), though the latter not surviving correction for multiple comparisons (adjusted alpha level set at.05/8 =.006). Correlation coefficients are reported in Table 3 (Column 1). Means and standard deviations according to age groups are reported in Supplementary Materials (Table S2).

**Table 3:** Column 1 shows Spearman correlation coefficients between subjective passage of time over the past year and age (in years), total Tele-GEMS score, and autobiographical memory retrieval over the past year

Variable	*M*	*SD*	1	2
1. How fast did the past year pass for you?	3.80	0.93	—	
2. Age	49.85	18.90	.08	—
3. Tele-GEMS	88.25	7.39	−.18	−.49***
4. Number of recalled memories	8.24	3.76	−.04	−.33***
5. Positive then	8.23	1.35	.27**	.18*
6. Positive now	8.51	1.12	.23*	.07
7. Significant then	4.14	0.75	.03	.30***
8. Significant now	4.00	0.85	−.04	.28**
9. Vividness	4.48	0.61	−.07	.26**

Column 2 shows Spearman correlation coefficients between age (in years), total Tele-GEMS score, and autobiographical memory retrieval over the past year. *p <.05. **p <.01. ***p <.001 (two-tailed)

The correlation difference between age and the subjective speed of time passage over the past 10 years and between age and the subjective speed of time passage over the past year was further tested using the R package *cocor* (Version 1.1–3; Diedenhofen & Musch, 2015) for the comparison of two overlapping correlations based on dependent groups (one-sided). The comparison was statistically significant across the large majority of tests (i.e., Dunn & Clark’s, 1969, *z*, *z* = 1.8537, *p* =.0319; Hendrickson et al., 1970, *t* = 1.8732, *df* = 117, *p* =.0318; Hittner et al., 2003, z = 1.8479, *p* =.0323; Hotelling’s, 1940, *t*, *t* = 1.8732, *p* =.0318; Olkin’s, 1967, *z*, *z* = 1.8849, *p* =.0297; Pearson & Filon’s, 1898,* z*, *z* = 1.8849, *p* =.0297; Steiger, 1980, *z* = 1.8482, *p* =.0323; Williams’s, 1959, *t, t* = 1.8698, *p* =.032); only the results of the test proposed by Zou (2007) did not support the hypothesis of a significant difference between correlations, since the 95% confidence interval for the difference [− 0.0103, 0.3655] included zero.

To explore age-related differences in autobiographical memory recall and cognitive functioning, Spearman correlation coefficients were further calculated between age, total Tele-GEMS score, and the number and subjective ratings of recalled memories over the past 10 years and over the past year.

**Fig. 2:**
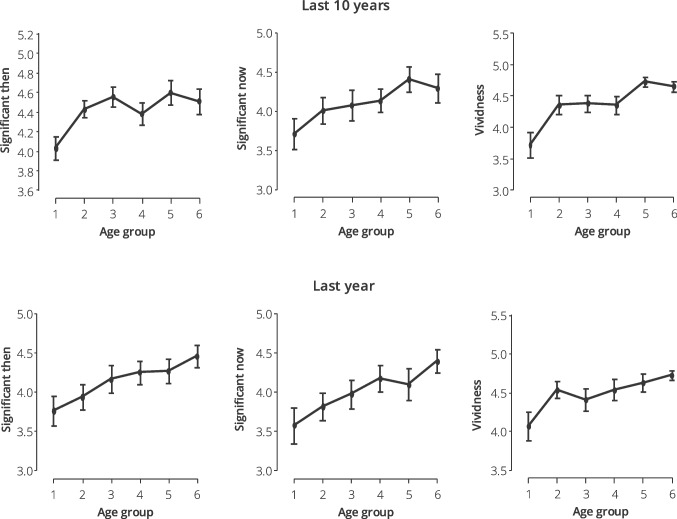
Means and standard errors over the different age groups for significance and vividness ratings of recalled events. 1 = age group 20–29; 2 = age group 30–39; 3 = age group 40–49; 4 = age group 50–59; 5 = age group 60–69; 6 = age group > 70

Participants’ age correlated negatively with the total Tele-GEMS score (*p* ≤.001) (Fig. 1c) and with the number of recalled memories both over the past 10 years (*p* <.001; Fig. 1d) and the past year (*p* <.001; Fig. 1e). Across both the past 10 years and the past year periods, age correlated positively with self-reported significance of events, as well as their vividness (all *p* values <.006; Fig. 2). Correlation coefficients are reported in Table 2 and Table 3 (Column 2).

### Cognitive factors associated with perceived time passage

As a second step of analyses addressing our confirmatory hypotheses on the association between age, experienced speed of time and physiological cognitive decline, a mediation analysis was performed to assess whether the association between age (X) and a faster passage of time over the past 10 years (Y) was partly accounted for by cognitive status (M), as measured by the total Tele-GEMS score.

Mediation analyses were performed using the R package *robmed* (Alfons et al., 2022), which applies the fast-and-robust bootstrap methodology for robust regression estimators, allowing it to deal with violations of normality assumption. We used 95% confidence level and 5,000 bootstrap replicates.

**Fig. 3:**
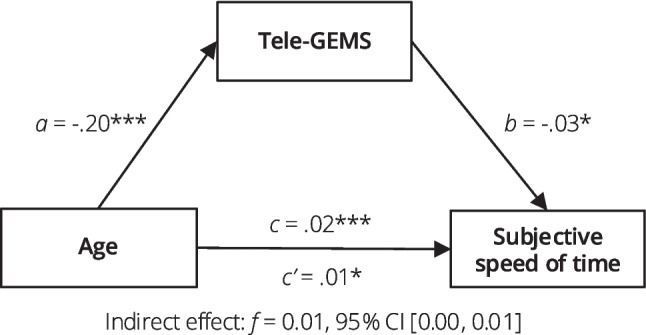
Relation between age, cognitive functioning as assessed by total score on the Tele-GEMS, and subjective speed of time over the past 10 years. a Path represents the direct effect of X (age) on M (Tele-GEMS). b Path represents the direct effect of M (Tele-GEMS) on Y (subjective speed of time). c Path represents the total effect of age on the subjective speed of time. ***c’*** path represent the direct effect of age on the subjective speed of time, controlling for cognitive functioning (Tele-GEMS). *p <.05. ***p <.001

Results are reported in Fig. 3. Age was significantly associated with cognitive status, with older participants reporting lower total Tele-GEMS scores. When controlling for age, the total Tele-GEMS score was significantly associated with the perceived speed of time over the past 10 years. The total effect of age on the perceived speed of time was significant, as well as the direct effect of age on the perceived speed of time, controlling for the total Tele-GEMS score. The indirect effect of age on perceived time passage was also significant, indicating partial mediation. Thus, while older age was associated with faster perceived time passage over the past 10 years, variations in cognitive functioning were also related to this pattern, as individuals with lower Tele-GEMS score tended to report greater time contraction.

### Cognitive domains underlying the perceived faster passage of time in older adults

Following the literature which consistently shows a decline of memory and executive functions in aging, and its effect on time perception (Maaß et al., 2019; Mioni et al., 2020), possible neuropsychological subdomains associated with the effect of global cognitive functioning on a faster subjective passage of time in older adults were also explored.

Spearman correlations coefficients were calculated between scores on items assessing the subjective speed of time over the past 10 years, participants’ age, and the Immediate memory, the Delayed memory, the Backward months and the Verbal fluency subtests of the Tele-GEMS. As a control analysis, the same correlations were calculated for the subjective speed of time over the past year.

In the Immediate memory subtest, assessing verbal short-term memory, participants are required to repeat six words right after the examiner. The Delayed memory subtest assesses participants’ ability to recall the same six words, requiring them to retrieve them after a delay of a few minutes. The Backward months subtest assesses working memory, asking participants to list the months of the year backwards, starting from October and skipping every other month. The Verbal fluency subtest assesses executive functions, requiring to select and follow a rule to access and produce as many words as possible in one minute, starting with the letter “T” and excluding proper names (see Montemurro et al., 2023, for a comprehensive description of the different subtests included in the Tele-GEMS).

Immediate memory recall performance significantly correlated with age (*p* <.001), but not with subjective time passage over the past 10 years (*p* =.07) or the past year (*p* =.19). Delayed memory performance was significantly correlated with age (*p* <.001), as well as with the subjective speed of time over the past 10 years (*p* =.001) but not over the past year (*p* =.07). Working memory scores and verbal fluency did not correlate significantly with the perceived speed of time over the past year (working memory: *p* =.08: verbal fluency: *p* =.15) or the past 10 years (working memory: *p* =.43; verbal fluency: *p* =.23), nor with age (working memory: *p* =.49; verbal fluency: *p* =.09). Correlation coefficients and descriptive statistics for the subtests are reported in Table 4.

**Table 4:** Column 1 shows Spearman correlation coefficients between age (in years) and subjective passage of time over the past ten years, subjective passage of time over the past year, and scores in the immediate memory, delayed memory, backwards months and verbal fluency subtests of the Tele-GEMS

Variable	*M*	*SD*	1	2	3
1. Age	49.85	18.90	—	—	—
2. How fast did the last 10 years pass for you?	3.55	1.00	.26**	—	—
3. How fast did the past year pass for you?	3.80	0.93	.08	.44***	—
4. Immediate memory	4.60	1.21	−.43***	−.17	−.12
5. Delayed memory	3.35	1.51	−.45***	−.29**°	−.17
6. Backwards months (working memory)	4.87	0.43	−.06	−.07	−.16
7. Verbal fluency (executive functions)	13.31	4.89	−.15	−.11	−.13

Column 2 and Column 3 show, respectively, Spearman correlation coefficients between subjective passage of time over the past 10 years and subjective time passage over the past year and the other variables. **p <.01; **°*p* =.001; ***p <.001 (two-tailed)

Finally, we performed a mediation analysis to examine the association between age (X), scores in the Delayed memory subtest of the Tele-GEMS (M), and the perceived speed of time over the past 10 years (Y).

**Fig. 4:**
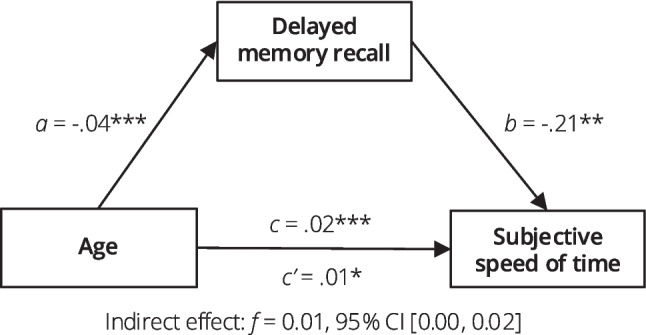
Relation between age, memory recall as assessed by the delayed memory subtest of the Tele-GEMS, and subjective speed of time over the past ten years. a Path represents the direct effect of X (age) on M (delayed memory recall). b Path represents the direct effect of M (delayed memory recall) on Y (subjective speed of time). c Path represents the total effect of age on the subjective speed of time. c’ Path represent the direct effect of age on the subjective speed of time, controlling for delayed memory performance. *p <.05. ***p <.01. *p <.001

Results are shown in Fig. 4. The direct effect of age on Delayed memory was significant, with reduced recall performance with increasing age. The effect of Delayed memory performance on the perceived speed of time over the past 10 years was also significant, with lower delayed recall scores corresponding to faster subjective time. The total and direct effect of age on the perceived speed of time were both significant, as well as the indirect effect of age on perceived time passage, indicating that the effect is partially mediated by the reduction in the ability to encode new information.

## Discussion

We examined potential factors underlying the well-documented phenomenon—and commonly held belief—that time seems to pass more quickly when one ages. More in detail, we directly tested the hypothesis that faster perceived passage of time in older adults is associated with the retrieval of fewer autobiographical episodes, leading to the impression of time contraction. Previous literature typically reported this effect for long (from 3 to 10 years), but not for relatively shorter time spans (e.g., the previous week, month, or year; Droit-Volet & Wearden, 2015; Friedman & Janssen, 2010; Gagnon-Harvey et al., 2021; Janssen et al., 2013; Winkler et al., 2017; Wittmann & Lehnhoff, 2005; Wittmann et al., 2015). Thus, to evaluate the specificity of the effect, we focused our investigation on two life periods—namely, the past 10 years and the past year.

For each of these time periods, we asked participants to retrieve as many autobiographical episodes as possible, within 90 s (Conti et al., 2024). Emotional valence, significance and vividness of events retrieved for the past 10 years and the past year were also assessed. Finally, based on evidence of an impact of age-related cognitive decline on time perception (Baudouin et al., 2019; Lamotte & Droit-Volet, 2017; Maaß et al., 2019; Mioni et al., 2020; Pütz et al., 2012a, 2012b; Ulbrich et al., 2009), we tested the association between global cognitive functioning and the perceived speed of time over the past 10 years and the past year.

We replicated the finding of a moderate correlation between age and the experienced speed of time over the past 10 years, but not over the past year (Droit-Volet & Wearden, 2015; Friedman & Janssen, 2010; Janssen et al., 2013; Wittmann & Lehnhoff, 2005). However, contrary to predictions, we found no relation between the subjective speed of time over the past 10 years and the number of autobiographical memories retrieved for the same time period.

Moreover, none of the assessed features of retrieved episodes (i.e., valence, personal significance and vividness) was associated with the experienced speed of time over the past 10 years. Previous studies reported inconsistent findings concerning the impact of significance and emotional valence of memories on subjective passage of time judgments for long time spans. Whereas Kosak and colleagues (2019) found that reporting more emotional and subjectively influential memories did not correspond to variations in the perceived speed of time over the past 5 years, a following study showed that passage of time judgments over the same period were associated with the retrieval of more impactful memories (Kosak & Kuhbandner, 2021). These studies involved participants on average younger than those included in the present sample, and explicitly required them to recollect, and describe in detail, positively or negatively valanced autobiographically relevant events (Kosak & Kuhbandner, 2021; Kosak et al., 2019). Our findings suggest that, when autobiographical memory retrieval is unconstrained, the phenomenon of faster time passage over long time spans and how it relates with age are not directly associated with the emotional valence or significance of the events.

A different pattern was observed for the experienced passage of time over the past year, which reported speed was significantly and positively correlated with the subjective positive valence of memories, especially at the time the events occurred. Thus, the more the autobiographical events over the past year were rated to have had a positive valence at the time they happened, the faster time over the same period seemed to have passed in retrospect. Shorter retrospective time estimates have been reported for stimuli with a positive emotional valence, and this effect was amplified when participants were paying attention to their bodily sensations (Pollatos et al., 2014). These findings have been linked to the role of bodily signals associated with emotion processing in shaping the perception of time (Wittmann & Droit-Volet, 2025), possibly also involving the retrieval of interoceptive and emotional states from memory (Teghil, 2025). From this perspective, retrieving many positively valanced episodes, during which time was experienced to pass fast, might have led to an overall retrospective impression of faster time passage over the past year.

A further finding concerns the positive association between participant’s age and subjective vividness and significance of reported events, both over the past 10 years and the past year. Older age is generally associated with reduced retrieval of episodic details (Levine et al., 2002; Martinelli et al., 2013; Piolino et al., 2006). However, the phenomenology of retrieved life memories does not necessarily become poorer with older age, and older adults might experience their memories as more vivid and significant than younger participants (Comblain et al., 2005; Janssen et al., 2011; Luchetti & Sutin, 2018; Rubin & Berntsen, 2009). When retrieval is unconstrained (i.e., no specific cue is provided for retrieval), older adults may select especially accessible, vivid and self-relevant memories (Comblain et al., 2005; Luchetti & Sutin, 2018), explaining the seemingly contradictory finding of higher rating of phenomenological features of memories, in spite of reduced production of episodic details (e.g., Levine et al., 2002).

A mediation analysis further highlighted that participants’ age and Tele-GEMS scores were both associated with the experienced speed of time. Lower scores on the Tele-GEMS, as well older age, corresponded to faster subjective time passage, specifically for the past 10 years. When considering different cognitive domains, we found that performance in the delayed memory recall subtest of the Tele-GEMS was the only measure associated with the experienced speed of time over the past 10 years. Lower delayed memory scores corresponded to faster subjective time passage, specifying the relation observed with global cognitive functioning.

Neuropsychological tests such as the delayed memory subtest of the Tele-GEMS, requiring to encode and retrieve newly presented lists of items devoid of any personal meaning, are routinary used to assess episodic memory ability—that is, the capacity to learn new item-specific information. Episodic memory is considered a basic component of complex event representations that characterize autobiographical memories, since representations of meaningful, self-relevant events, experienced in everyday life, involve the interplay between a large set of processes, including self-reflection, executive functions, and semantic processing (Grilli & Sheldon, 2022; Renoult et al., 2012; Svoboda et al., 2006).

The present findings that delayed memory recall ability, but not autobiographical memory, is associated with faster subjective time passage over the past 10 years with increasing age, suggest that this impression may be related to reduced encoding of new episodic information, rather than to reduced availability of memories of personal life events. It is possible that the impression of how fast the past 10 years have passed is associated not as much with how many personal events can be readily accessed at the time of the judgment, but rather with a more general impression of how much memory encoding has taken place within the 10-year time interval.

Episodic memory encoding is usually reduced in aged individuals, with the decrease in learning happening gradually over the adult lifespan (Balota et al., 2000; Cansino, 2009). If such a decline in episodic learning is relatively constant over time, older adults may experience the impression of overall reduced memory content over long time intervals, such as the past 10 years, leading to a perceived acceleration of time during the same intervals. It has been proposed that, due to an age-related slowing in the frequency of sensory sampling, older adults experience a reduction in the density of perceived events, which would result in lower memory encoding and in the retrospective impression of a faster time passage (Bejan, 2019). This interpretation is consistent with the hypothesis that, due to the progressive reduction in the average storage of new event information, older adults may perceive a mismatch between predicted and experienced amount of memory storage, leading to the retrospective impression that time has passed comparatively faster (Wearden, 2005).

From this perspective, the more pronounced effect of aging on the perceived speed of time over relatively longer compared with shorter time periods (such as the 10-year and 1-year intervals tested in the present study) might result from the different proportions of these intervals relative to the entire lifespan. The principal idea that the subjective experience of time is related to the proportionality between a judged time interval and one's age has been first formulated by Paul Janet in 1877 (Wearden, 2021). The proportion of one year relative to the entire lifespan is small (e.g., 1/40, 1/80). Thus, the difference in the average encoding of new information over a single year could be relatively imperceptible. In contrast, when considering a 10-year span, the larger proportion of the interval with respect to the entire lifespan (e.g., 10/40, 10/80) would be more salient, allowing older adults to detect a mismatch between expected and perceived storage of new events, with the result of a retrospective impression of a faster time passage. Although this is an appealing interpretation, further studies would be needed to directly test this hypothesis, possibly combining measures of cognitive processing with a more in-depth characterization of strategies and heuristics involved in estimating the perceived speed of time.

Notably, a quadratic component was apparent in the association between age and the subjective speed of time over the past 10 years, suggesting that the perceived speed of time over the past decade increases with age up to a point, after which the rate of increase levels off, or slightly declines. This is in line with evidence that the effect of age on the perceived speed of time over the past 10 years may reach a plateau at around the age of 60 (Wittmann & Lehnhoff, 2005). Moreover, we highlighted only a partial mediation effect of cognitive functioning and episodic memory encoding between older age and faster subjective time over the past 10 years. Taken together, these results suggest that aging may account only partially for differences in the perceived speed of time over the past decade, while other factors might be at play. Previous research has shown that the experienced time pressure, the number of routines and new experiences in everyday life (Winkler et al., 2017), the time perspective and emotion regulation skills (Wittmann et al., 2015), and, possibly in relation with these same factors, the number of children (Wittmann & Mella, 2021), all affect the perceived speed of time on relatively long time spans of several years. Future studies could be adopting an integrated approach, jointly assessing cognitive, personality and social factors possibly contributing to the impression that the past years feels to move faster.

It is also important to point out that, in the present study, the exclusion of the youngest age group from analyses significantly impacted the results. This suggests that the overall age effect in our sample was at least partly driven by the difference between the youngest age group and the other age groups. Previous studies involving larger samples suggest that the association between age and subjective time speed involves a lifespan trajectory (Friedman & Janssen, 2010; Gagnon-Harvey et al., 2021; Winkler et al., 2017). The large impact of ratings provided by the youngest age group in the current data is likely associated with the relatively small sample size, suggesting some caution in generalizing the results. Nonetheless, a pattern suggestive of a tendency for very young individuals to experience time over the past 10 years as having passed especially slow is also apparent in some previous studies (Friedman & Janssen, 2010; Fig. 2; Wittmann & Lehnhoff, 2005). Several findings have linked differences in prospective time perception across the development (i.e., from childhood to adolescence) to changes in cognitive processes, such as executive control and memory (see Buzi et al., 2024, for a review). Nonetheless, to the best of our knowledge, the retrospective experience of time in young and very young adults has not been systematically investigated. It is thus unclear if the cognitive factors mentioned above may affect retrospective passage of time judgments in this population. Other factors, such as those related to the number of new life experiences and the experienced time pressure (Winkler et al., 2017) may also have a significant impact on perceived time passage during these stages of life. Given the relatively small number of participants included in each age group in the present study, our data are not conclusive regarding this issue. Nonetheless, tracking possible variations in the retrospective experience of time and their underlying mechanisms across the lifespan might be a promising direction for future studies.

Further limitations should be acknowledged. First, a significantly stronger correlation was highlighted between age and the perceived speed of time passage over the past decade versus the past year. Nonetheless, a more conservative estimate based on confidence intervals suggested that this difference might actually be modest. Studies in larger samples are thus needed to provide more reliable estimates of differential age effects on the subjective speed of time over different time spans. Second, an overall reduction in processing speed with older age has been proposed as a key factor in explaining age-related effects on the experienced speed of time (Baudouin et al., 2019; Christensen, 2001; Pütz et al., 2012a, 2012b). Since processing speed was not directly assessed here, it is not possible to exclude this factor had an influence on observed results. Assessing processing speed and its reductions with aging would allow us to understand if such an effect might be dissociated by that linked with general cognitive decline, and its specific contribution to age-related changes in the perception of time passage.

Although the present results support the association between age and faster perceived time passage over the past decade, potential alternative explanation should also be taken into account. For example, considering the wide-spread belief about faster subjective time speed in aging, it is possible that older individuals may conform to this expectation, providing higher ratings in surveys assessing this phenomenon. Future studies could control for this possibility explicitly including a demand characteristics survey at the end of the experimental procedure.

Finally, an intrinsic limitation of the present study lies in the cross-sectional nature of data, which does not allow us to draw causal inferences on mechanisms contributing to possible changes in subjective time over the lifespan (see, e.g., Lindenberger et al., 2011, for a discussion of this issue). Nonetheless, present results show that the perception of faster time passage with increasing age is not explained by autobiographical memory recall, but rather associated with reduced cognitive efficiency, and specifically with the ability to encode new information, supporting the investigation of episodic memory encoding as a candidate factor in explaining differences in the perception of time passage and their possible changes over the lifespan.

## Supplementary Information

Below is the link to the electronic supplementary material.

Supplementary file1 (PDF 73 kb)

## Data Availability

The dataset used in all reported analyses may be accessed at the link https://osf.io/t65k9/?view_only=25bf94f7cca6481fb1cbc0b03d9e0c9c

## Code availability

All analyses were performed using standard statistical software.
