# Supplementary material for: Memory encoding for new information, not autobiographical memory load, is linked with age-related differences in subjective time passage over the past decade
Source: Mem Cognit. 2026 Apr 7;54(6):2394–408. doi: 10.3758/s13421-026-01879-1 (PMC13461740; doi:10.3758/s13421-026-01879-1)
Supplement: Supplementary file 1 [file 13421_2026_1879_MOESM1_ESM.pdf]

Supplementary materials

File S1. Full list of items included in the Subjective Time Questionnaire (STQ).

Rating scale:

1	2	3	4	5
Very slowly	Slowly	Neither fast nor slow	Fast	Very fast

Present[1]

1. How fast does time usually pass for you?
2. How fast do you expect the next hour to pass?

Past[1]

3. How fast did the previous week pass for you?
4. How fast did the previous month pass for you?
5. How fast did the previous year pass for you?
6. How fast did the previous 10 years pass for you?
7. How fast did your childhood (< 12 years) go by?
8. How fast did your youth (13-19 years) go by?
9. How fast did your adulthood between 20 and 29 years go by?
10. How fast did your adulthood between 30 and 39 years go by?

Rating scale:

1	2	3	4	5
Strongly disagree	Disagree	Neither agree nor disagree	Agree	Strongly agree

Time pressure[1]

11. I haven't enough time to complete my tasks.
12. I often feel time pressure.
13. I often haven't enough time to devote myself to important things.
14. I often think time is running out.
15. I have to establish my priorities, because I cannot do all the things I would like to do.

Time expansion[1]

16. My time seems empty.
17. I often think that time just does not want to pass.
18. I often feel bored.
19. I have a lot of time.
20. I often have spent my time without doing anything.

Metaphors: Speed[1]

21. Time is a speeding train.
22. Time is a galloping horse.
23. Time is a tumbling waterfall.

Metaphors: Slowness[1]

24. Time is a vast expanse of sky.
25. Time is a quiet, motionless sea.
26. Time is a tedious song.

Experience of recent life changes[2]

27. The past two years have been a time filled with many new experiences (reverse scored).
28. In the past several years, my life has been quite routine.
29. When I think back over the past two years, few notable events come to mind.
30. There have been few notable changes in my life in the past year.

Forward telescoping[2]

31. When I try to remember the date of some event, I often come up with a time that is not as long ago as the true time.
32. When I think that something was just a few years ago, it often turns out that it happened long before that.
33. I often find that things actually occurred much longer ago than I thought (reverse scored).

[1] Items from Wittmann and Lehnhoff (2005).
[2] Items from Friedman and Janssen (2010).

References

Friedman, W. J., & Janssen, S. M. (2010). Aging and the speed of time. Acta Psychologica, 134(2), 130–141. https://doi.org/10.1016/j.actpsy.2010.01.004

Wittmann, M., & Lehnhoff, S. (2005). Age effects in perception of time. Psychological reports, 97(3), 921–935. https://doi.org/10.2466/pr0.97.3.921-935

Table S1.

		M	SD	*W*	*p*
Last 10 years	Positive then	7.87	1.37	1234	.031
	Positive now	7.98	1.400		
	Significant then	4.41	0.535	1680	< .001
	Significant now	4.10	0.800		
	Vivid	4.36	0.700		
Last year	Positive then	8.23	1.35	539	< .001
	Positive now	8.51	1.12		
	Significant then	4.14	0.75	1172	< .001
	Significant now	4.00	0.853		
	Vivid	4.48	0.610		

Descriptive statistics (mean and standard deviation) of qualitative ratings for retrieved autobiographical memories in the Autobiographical Fluency Task over the last 10 years and the last year periods. For each period, results of Wilcoxon W tests comparing positivity and significance ratings between the time of the event ("then") and the present moment ("now") are also reported.

Table S2.

	Age group											
	1		2		3		4		5		6	
**Variable**	**M**	**SD**	**M**	**SD**	**M**	**SD**	**M**	**SD**	**M**	**SD**	**M**	**SD**
Tele-GEMS	92.97	4.60	90.98	4.89	88.76	6.17	89.80	5.39	87.28	6.98	79.69	8.29
How fast did the last 10 years pass for you?	2.85	0.88	3.80	1.01	3.45	1.05	3.70	0.87	3.85	0.67	3.65	1.18
Last 10 years - Number of recalled memories	10.1	2.42	10.6	4.66	9.50	3.12	8.80	3.44	8.35	3.44	5.30	2.66
Last 10 years - Positive then	7.69	0.90	7.69	1.20	8.20	1.16	7.52	1.50	8.34	1.74	7.77	1.52
Last 10 years - Positive now	8.05	1.02	7.87	1.24	8.40	0.96	7.48	1.41	8.46	1.69	7.67	1.76
Last 10 years - Significant then	4.03	0.55	4.43	0.37	4.55	0.47	4.38	0.52	4.59	0.56	4.50	0.57
Last 10 years - Significant now	3.71	0.86	4.01	0.76	4.07	0.88	4.13	0.68	4.41	0.72	4.30	0.80
Last 10 years - Vivid	3.71	0.90	4.35	0.70	4.37	0.62	4.35	0.66	4.72	0.35	4.64	0.39
How fast did the last year pass for you?	3.5	1.10	3.85	0.67	3.95	0.95	3.75	0.85	4.15	0.75	3.60	1.14
Last year - Number of recalled memories	9.55	3.35	9.5	3.80	7.85	2.91	8.25	3.85	8.20	3.44	6.10	4.38
Last year - Positive then	7.8	0.99	8.29	1.15	8.25	0.99	7.97	1.75	8.75	1.12	8.32	1.80
Last year - Positive now	8.37	0.89	8.51	1.13	8.63	0.90	8.25	1.14	8.76	1.06	8.53	1.56
Last year - Significant then	3.76	0.84	3.93	0.71	4.16	0.80	4.25	0.68	4.26	0.68	4.46	0.65
Last year - Significant now	3.57	1.01	3.81	0.76	3.97	0.82	4.17	0.75	4.09	0.92	4.39	0.67
Last year - Vivid	4.06	0.82	4.54	0.51	4.41	0.62	4.54	0.61	4.62	0.53	4.73	0.28

Descriptive statistics (mean and standard deviation) of assessed variables over the different age groups. 1 = age group 20-29; 2 = age group 30-39; 3 = age group 40-49; 4 = age group 50-59; 5 = age group 60-69; 6 = age group > 70.

Table S3.

Variable	M	SD	1	2	3	4	5	6
1. How fast did the last 10 years pass for you?	3.55	1	--					
2. Positive then	7.71	2.04	.11	--				
3. Positive now	7.94	1.88	.11	.891**	--			
4. Significant then	4.48	0.62	-.180*	.09	.09	--		
5. Significant now	4.22	0.81	-.09	.11	.05	.726**	--	
6. Vivid	4.37	0.78	.07	.05	.04	.372**	.522**	--

Spearman correlation coefficients between subjective passage of time over the last ten years and autobiographical memory retrieval over the same time period, considering only the first three items reported in the Autobiographical Fluency Task. *p < .05. **p < .01. ***p < .001 (two-tailed).
